# A guide for a patient-centric approach to asthma management: results of a European Delphi consensus programme

**DOI:** 10.1038/s41533-025-00465-3

**Published:** 2025-12-19

**Authors:** Fulvio Braido, Ilaria Baiardini, Simona Barbaglia, Susanna Palkonen, Armando Ruiz, Ioanna Tsiligianni, Johann Christian Virchow, Tonya Winders

**Affiliations:** 1 RCCS Ospedale Policlinico San Martino, Genoa, Italy; Università di Genova, DiMI, Genoa, Italy; 2 Respiratory Diseases and Allergy Department, Department of Internal Medicine, University of Genoa, Genoa, Italy; 3 Associazione Nazionale Pazienti Respiriamo Insieme APS, Padova, Italy; 4 European Federation of Allergy and Airways Diseases Patients Associations (EFA), Stockholm, Sweden; 5 Spanish Federation of Allergy and Airways Diseases Patients’ Associations (FENAER), Alicante, Spain; 6 Clinic of Social and Family Medicine, Department of Social Medicine, Faculty of Medicine, University of Crete, Rethymno, Greece; 7 Department of Primary Care and Population Health, Medical School, University of Nicosia, Nicosia, Cyprus; 8 Departments of Pneumology, Intensive Care Medicine, University of Rostock, Rostock, Germany; 9 Global Allergy & Airways Patient Platform (GAAPP), Tennessee, USA

**Keywords:** Diseases, Health care, Medical research

## Abstract

*Background:* The Global Initiative for Asthma 2024 report recommends a shared decision-making approach to guide treatment choice, encompassing patients’ goals, beliefs and concerns about asthma and medications (GINA. Global Strategy for Asthma Management and Prevention, 2024). There is limited guidance on ways to achieve this goal. This consensus programme aimed to create recommendations on optimal selection of inhaler treatment while considering patient perspectives and needs. *Methods:* A literature review was conducted on literature published between 01/01/2014 and 23/04/2024 using agreed keywords and search parameters in PubMed and Cochrane databases. Evidence on impact of patient factors on adherence and asthma control, plus inhaler preference data, was analysed. A consensus voting panel was selected via screening questionnaire, with 50 patients with asthma duration ≥5 years and 39 healthcare professionals with expertise in asthma from five European countries (Germany, France, Czechia, Italy, Greece). A two-round Delphi method was used. *Results:* 40/135 papers were considered relevant. From these, 20 consensus statements were developed in four areas: patient-centred treatment selection, medication/asthma beliefs, patient preference + shared decision-making, and tools for patient-centred care. 18/20 consensus statements were accepted with an agreement threshold >85% on the first round of voting. Two revised statements underwent a second Delphi round, again failing to reach consensus. *Conclusions:* This important initiative generated much-needed guidance on integrating patient views and needs into treatment decision-making following a well-established methodology through 18 consensus statements, with nearly equal input from patients and healthcare professionals.

## Introduction

Successful asthma management requires treatment of both symptoms and underlying pathology, and inhaled therapies remain the mainstay of treatment^1^. As asthma is a lifelong condition, it is essential to select not only the right drug but also appropriate long-term holistic care. Medication adherence plays a key role in the success of any treatment and, for this reason, a patient-centred approach to treatment selection can be beneficial since patients are more likely to adhere to their medication when they play a part in decision-making about their care^2,3^. The ‘right’ approach is affected by multiple factors, including the patient’s age, literacy, health literacy, physical abilities, comorbidities, lifestyle, perceived stigma, and beliefs about their condition and medications^2,4–15^.

Shared decision-making – involving the patient in decisions about their treatment – may be viewed as an ethical obligation as well as a means to improve medication adherence. This approach is being increasingly adopted in the management of many chronic health conditions, with varying degrees of success^16–20^. In asthma, the details of what shared decision-making and personalising treatment look like in practice are not well defined^1,3,21^. Guidelines provide broad recommendations without clear direction on their implementation, leaving the details of how to put recommendations into practice open to interpretation. This has led to various approaches with variable outcomes. A systematic review of the literature on shared decision-making in asthma revealed that whilst some studies reported benefits of shared decision-making on outcomes like patient quality of life and exacerbations, other studies reported no benefit when measured by the same outcomes^2,22^. Thus, more clarity is needed on how healthcare professionals (HCPs) should engage with patients to determine an appropriate shared decision-making approach to asthma management^23^.

In light of the need for more practical guidance, a consensus to guide HCPs in adopting a patient-centric approach to asthma management was sought. The aim was to provide guidance on optimal treatment selection, incorporating patients’ preferences, beliefs, behaviours, needs and attitudes alongside the HCPs’ guidance. The Delphi methodology, an iterative process used to solicit and distil the judgments of experts using a series of questionnaires alternated with feedback, is widely used to gain consensus in areas of medical unmet need, and supports content validity through iterative group input and structured feedback^24–26^. The programme described here employed a modified Delphi method to reach consensus, using two rounds of anonymous online voting on statements drafted based on evidence retrieved in a literature search.

## Methods

### Panel formation

The voting panel comprised of a multidisciplinary steering committee (SC) and a larger consensus voting group. The SC members were recruited according to scientific background and expertise, activity in patient advocacy organisations, and/or online research. To ensure diversity in viewpoints and experiences, HCPs and patients (people with asthma) or patient advocates (those representing people with asthma) were recruited from Germany, Italy, France, Greece and Czechia, representing a range of varying healthcare models across Europe. The role of the SC was to guide the consensus approach, determine clinical questions, advise on formation of the voting panel, and draft and revise consensus statements.

The aim was to recruit 50 HCPs and 50 patients to the voting panel. HCPs who were highly experienced general practitioners, specialists and/or asthma nurses who were actively managing at least 10 patients with asthma were invited. The target patient profile included adults who have had asthma for at least 5 years and who currently use inhaler(s). Screening questionnaires were designed to identify participants meeting these criteria (Tables S1 and S2) and were distributed via specialist agencies (Knowledge Gate Group for HCPs and Exafield International for patients).

**Fig. 1:**
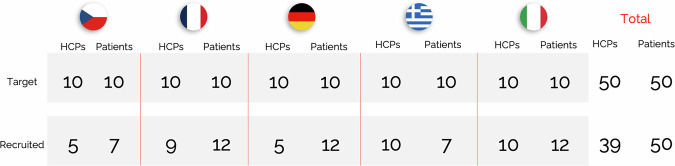
Formation of the voting panel. HCP, healthcare professional.

The SC consisted of eight members: four HCPs, two patient representatives and two representatives from asthma patient advocacy organisations. The final voting panel consisted of 89 participants: 39 HCPs and 50 patients (Fig. 1 and Table 1).

**Table 1:** Details of the 39 healthcare professionals recruited into the voting panel.

Location	Gender	Specialty	Primary setting	Years practicing medicine	Time seeing patients	Years managing patients with asthma	Number of patients/month	Published in asthma	Inhaler publications
Germany (*n* = 5)	M	GP	GP community clinic	11–20	>70%	16+	16+	Y	Y
	M	GP	GP community clinic	6–10	>70%	4–7	8–10	N	N
	M	GP	Multi-disciplinary community clinic	6–10	>70%	8–10	16+	N	N
	M	GP	Non-community/private	11–20	>70%	8–10	16+	N	N
	M	Internal Medicine	Multi-disciplinary community clinic	20+	>70%	16+	11–15	N	N
Italy (*n* = 10)	M	Allergist	Specialist respiratory clinic	20+	>70%	16+	16+	Y	Y
	M	GP	GP community clinic	11–20	>70%	11–15	11–15	N	N
	M	GP	GP community clinic	6–10	>70%	8–10	16+	N	N
	M	Allergist	Specialist respiratory hospital	11–20	51–70%	11–15	16+	N	N
	M	Pulmonologist/respiratory physician	Specialist respiratory hospital	20+	>70%	16+	16+	N	N
	M	Pulmonologist/respiratory physician	Specialist respiratory clinic	11–20	51–70%	8–10	4–7	N	N
	M	Allergist	Multi-disciplinary community clinic	6–10	>70%	8–10	16+	Y	Y
	M	Pulmonologist/respiratory physician	Multi-disciplinary community clinic	11–20	>70%	16+	16+	N	N
	M	GP	GP community clinic	6–10	>70%	8–10	16+	N	N
	F	Allergist	Specialist respiratory clinic	11–20	51–70%	8–10	16+	Y	N
Greece (*n* = 10)	F	Pulmonologist/respiratory physician	Specialist respiratory hospital	20+	>70%	16+	16+	Y	Y
	F	Pulmonologist/respiratory physician	Specialist respiratory hospital	11–20	>70%	11–15	16+	Y	Y
	M	Pulmonologist/respiratory physician	Specialist respiratory hospital	20+	51–70%	16+	16+	Y	Y
	M	Pulmonologist/respiratory physician	Specialist respiratory hospital	20+	>70%	11–15	11–15	Y	N
	M	Pulmonologist/respiratory physician	General hospital	11–20	>70%	16+	11–15	Y	N
	M	GP	Multi-disciplinary community clinic	20+	>70%	11–15	11–15	N	N
	M	Pulmonologist/respiratory physician	Specialist respiratory hospital	11–20	51–70%	11–15	4–7	N	N
	M	Allergist	Non-community/private	20+	>70%	16+	16+	N	N
	M	Pulmonologist/respiratory physician	General hospital	20+	51–70%	16+	16+	Y	N
	M	Pulmonologist/respiratory physician	Non-community/private	20+	>70%	16+	16+	Y	N
France (*n* = 9)	M	Pulmonologist/respiratory physician	Specialist respiratory hospital	6–10	>70%	11–15	16+	Y	Y
	M	GP	Non-community/private	20+	>70%	16+	16+	N	N
	M	Pulmonologist/respiratory physician	Specialist respiratory clinic	6–10	>70%	8–10	16+	N	N
	M	Pulmonologist/respiratory physician	Specialist respiratory hospital	20+	>70%	16+	11–15	N	N
	F	Pulmonologist/respiratory physician	Specialist respiratory hospital	6–10	>70%	4–7	16+	N	N
	F	Pulmonologist/respiratory physician	Non-community/private	20+	>70%	16+	16+	N	N
	M	Pulmonologist/respiratory physician	Specialist respiratory hospital	11–20	51–70%	11–15	11–15	Y	N
	F	Allergist	Non-community/private	11–20	>70%	11–15	16+	N	N
	NR								
Czechia (*n* = 5)	M	Pulmonologist/respiratory physician	Non-community/private	11–20	>70%	11–15	16+	N	N
	M	Allergist	Non-community/private	20+	>70%	16+	16+	Y	N
	F	Pulmonologist/respiratory physician	Specialist respiratory clinic	20+	>70%	11–15	16+	Y	Y
	M	NR							
	M	NR							

*GP* general practitioner, *HCP* healthcare professional, *NR* not reported.

Please note that details of one HCP from France are not included.

### Literature search

The draft statements were based on evidence gathered via a non-systematic literature search, performed in April 2024 using PubMed and the Cochrane Database of Systematic Reviews and restricted to adult patients. Search criteria and limiters are described in supplementary materials (Table S3). During the appraisal of the retrieved publications, duplicates were removed and publications identified as relevant to the programme objectives were included in an evidence summary output.

### Consensus voting

Statements developed during the Delphi process were organised into an online survey and distributed to participants via SurveyMonkey. All consensus statements were approved by the SC before submission to the voting panel for voting.

Participants voted on the statements in two rounds. They were asked to select their level of agreement with the draft consensus statements. They could choose one option per statement from a five-point scale (strongly agree, agree, neutral, disagree, strongly disagree). They were also asked to provide suggestions for improving any statements they disagreed with (to which they had answered ‘disagree’ or ‘strongly disagree’).

The SC reviewed comments from the voting panel and amended any statements that had not achieved consensus. Updated statements were then shared with the group for another round of voting. Results from this second round were considered final and the statements were classified as either accepted or not accepted. Statements that failed to achieve the required level of consensus were identified as non-consensus statements.

### Data analysis

The agreed threshold for consensus was >85%. The agreement level was calculated using the following formula:

$$\left(\frac{({Number}\,{of}^{\prime}\, {Strongly}\,{agree}^{\prime}\, +{Number}\,{of}^{\prime}\, {Agree}^{\prime} )}{{Number}\,of\,{responses}}\times 100\right)$$

This method is not weighted to reflect the level of agreement.

## Results

### Literature search

**Fig. 2:**
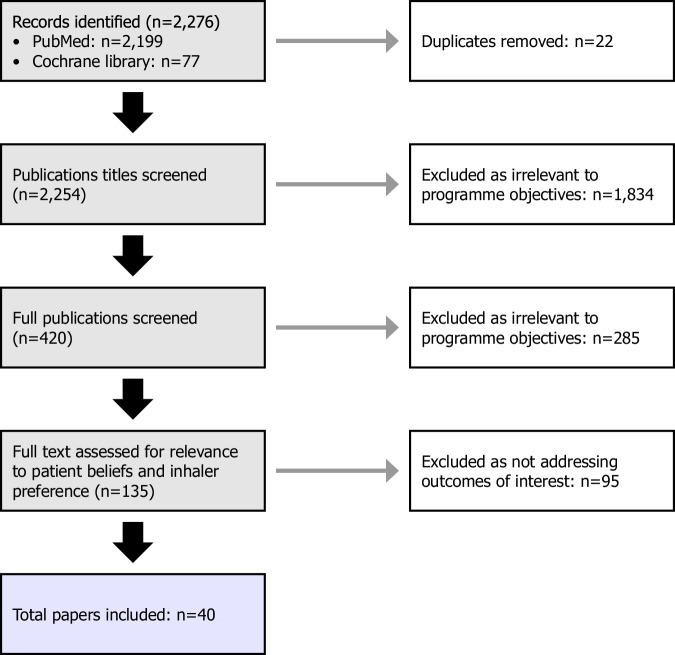
Flow diagram of the literature search and appraisal process.

A total of 40 publications were included in the final evidence summary (Fig. 2, Table S4).

### Voting

The SC reviewed the evidence summary output and identified four main themes of interest. Twenty consensus statements were drafted and grouped under four themes for the purposes of voting: patient-centred treatment selection (*n* = 4), medication and asthma beliefs (*n* = 4), patient preferences and shared decision-making (*n* = 9), and utilising tools and technology to support patient-centred care (*n* = 3).

In round 1, 80 of the 89 participants completed the survey (87% of HCPs [34/39] and 92% of patients [46/50]). Consensus was reached on 18 statements. There were two statements that did not reach consensus:

*‘Cultural beliefs can affect how patients feel about using an inhaler. Doctors/nurses should talk to their patients about this and make decisions together to choose the right treatment’* *.*

- Out of a total of 75 respondents, three HCPs and 12 patients did not agree with this statement (25%). Four disagreed that cultural beliefs influence or inhibit the use of inhalers, and an additional 12 responded ‘Neutral’.
*‘When choosing a treatment, doctors/nurses should think about the negative feelings and social stigma that can come with using an inhaler. Being embarrassed or worried can affect if a patient wants to use their inhaler regularly’* *.*
- For this second statement, nine out of a total of 75 respondents reported observing no stigma or feelings of embarrassment associated with inhaler use; all but one of these responses were from patients. Interestingly, all 12 German patients agreed with this statement, suggesting potential variation between countries on this topic. Four respondents disagreed with this statement because they believed that it is the patient’s responsibility to manage potential negative feelings, and not that of the HCP.

These two statements were revised by the SC, who agreed to consolidate them into a single lay-language version:

*‘When choosing a treatment, doctors and nurses should remember that some patients may feel embarrassed, face social stigma or have cultural beliefs that affect how they use an inhaler. These factors can affect treatment adherence’* *.*

In round 2 of the modified Delphi voting, 87 voting panel members completed the survey (97% of HCPs [*n* = 38/39] and 98% of patients [*n* = 49/50]). However, the revised statement failed to gain consensus, with the level of agreement below the specified threshold of 85% (67.82%).

After two rounds of voting, consensus was reached on 18 statements (Table 2). The level of consensus reached for each statement can be found in Table S5.

**Table 2:** Final recommendations.

Domains	Consensus statements
Patient-centred treatment selection	Treatment should fit a patient’s daily life and be personalised. Problems like cost, trouble using the inhaler and beliefs about asthma or inhalers need to be considered. This will help patients use their inhalers regularly and correctly.
	Doctors/nurses and patients should work together to find out how asthma affects the patient’s life. They should also discuss why asthma control is important for their short- and long-term goals of treatment. This will make sure that patients use their inhaler regularly and are happy with it.
	Correct inhaler technique and the ability to breathe in strongly are key for deciding between dry powder inhalers (DPIs) and metered-dose inhalers (MDIs). DPIs are not right for patients who can’t inhale strongly enough.
Medication and asthma beliefs	Patients’ understanding of asthma and why they need an inhaler can differ. Doctors/nurses should check what patients know about why they need treatment and give them the right information and education if needed. This helps choose the best treatment and encourages regular use.
	What patients know, believe and have experienced about how well asthma inhalers work and how safe they are can affect if they use them as suggested. Doctors/nurses should ask patients to share their thoughts and worries about inhalers, so any confusion or concerns can be cleared up.
	Any reasons why a patient might not follow their treatment should be discussed openly. A team approach helps find and fix any problems, leading to better asthma control.
Patient preference and shared decision-making	Including patients in decisions about their asthma treatment can lead to better results. It can help them stick to their treatment if it is right for them, and it fits their needs and lifestyle.
	How easy the inhaler is to use and the right technique can affect if a patient is satisfied and follows their treatment. Doctors/nurses should provide training and agree with their patient on the right device for them.
	Choosing the right inhaler should consider both the medicine and practical things, like portability and a dose counter. This helps make sure the treatment fits the patient’s needs in both aspects.
	Some patients prefer using just one inhaler instead of two or more. Doctors/nurses should take this into account to plan a patient’s treatment. It can help to improve patient satisfaction and make it easier for them to stick to their treatment.
	Some patients have concerns about non-branded inhalers compared with brand name ones. Doctors/nurses should address these concerns when they choose a non-branded inhaler to make sure patients follow their treatment.
	Doctors/nurses should teach their patients about different inhalers, how to use them, and their risks and benefits. This helps to decide on a treatment together and can improve how well patients follow their treatment.
	Doctors/nurses and patients should agree on what good asthma control looks like and how to reach it. This shared understanding of treatment goals helps with better asthma care.
	Treatment goals should be reviewed at each visit to make sure both the patient and provider are working towards the same goals.
	Both the doctor/nurse and patient should agree on a treatment plan. This makes sure the plan meets the patient’s needs, leading to better results and regular use of the treatment.
Utilising tools and technology to support patient-centred care	Doctors/nurses can use tools like the RACE questionnaire to find out what might make it hard for a patient to stick to their inhaler treatment.
	There are many apps and tools that can help patients check if they are using their inhaler properly and often enough. Doctors/nurses should assess each individual patient and, if appropriate, suggest apps or tools that might help them.
	Patients should get treatment or self-management plans in writing, either electronically (so they can print them) or on paper.

*DPI* dry powder inhaler, *MDI* metered-dose inhaler, *RACE* respiratory adherence care enhancer.

## Discussion

In this consensus programme, a large and diverse group was able to gain consensus on 18 statements concerning patient-centred asthma management. The findings are a clear indication that HCPs and patients agree on patient-centred care as a positive approach to asthma management. The breadth of statements that reached consensus support a multifactorial and consistent approach to patient involvement throughout the patient’s treatment journey.

Every stage of the decision-making process in asthma treatment requires sharing of information between the physician and the patient. The consensus statements highlight the importance of first ascertaining the patient’s level of understanding about their asthma and why they need an inhaler, before giving them information appropriate for their knowledge level. Information gathering should also include how asthma affects the patient’s daily life, their expectations of treatment, and potential barriers to the patient adhering to their prescribed regimen^1^. Such information should be discussed openly with the patient. Only by following these important initial steps can the HCP then provide the patient with appropriate educational resources that they can access at home^1^, examples of which are provided by Asthma + Lung UK^27^, Global Allergy & Airways Patient Platform^28^, Allergy & Asthma Network^29^ and National Asthma Education Programme^30^.

HCPs must purposefully collect such information from the patient to inform decision-making. This may be by means of questionnaires and other tools, the results of which should be discussed with the patient during consultations. The consensus statements highlight the utility of the Respiratory Adherence Care Enhancer (RACE) questionnaire in patient-centred asthma management^31^. Other validated apps and tools, with different focal points, are available that may also be useful^32^. A key component is for the HCP to have an open and non-judgemental attitude towards the patient’s thoughts, emotions and behaviours, in order to garner patient trust and, therefore, honest information-sharing^33^. Misconceptions or concerns may lead to underuse or misuse of inhalers and other prescribed medications.

Armed with the right information, the HCP and patient should discuss the treatment plan, including appropriate goals and what ‘good’ asthma control means to them^1^. Personalised goal-setting can ensure that management is not only patient-centred but also practical and achievable^34^, especially when using a SMART (specific, measurable, achievable, relevant and time-bound) approach and when the patient is provided with a written action plan. Written asthma action plans (either electronic or paper-based) are strongly recommended by clinical guidelines^1^ due to their proven role in enhancing patient adherence and self-efficacy, and their role in improving outcomes^35^.

Trust-based relationships develop over time, and regular follow-up consultations are essential in patient-centred care^36^. They can be used to evaluate and review progress, including any barriers to compliance, changes in beliefs about the medication, and educational gaps^37^. With a wide variety of inhaler options available, the selection of the correct device plays a crucial role in achieving optimal asthma control^3,38,39^. For circumstances in which a patient might initially express no preference for an inhaler, time and experience may change this such that the patient prefers particular features, such as portability and a dose counter, or even the number of inhalers used^3,38^. Whilst repeat consultations place greater demands on the HCP’s time, this may be efficient over the long term due to increased patient satisfaction and adherence to medication.

These consensus statements may also challenge ‘normal’ behaviours and possible habitual prescribing habits. In some cases, statements about specific treatment approaches, such as the use of non-branded inhalers, multiple inhaler regimens and metered-dose inhalers versus dry powder inhalers, stimulate discussions between HCPs and patients. Such conversations may have a beneficial impact on treatment adherence and, subsequently, on patient outcomes. For example, addressing patient concerns about generic versus branded inhalers can be an easy but essential step in maintaining adherence and trust^40^. Clear communication and reassurance regarding therapeutic equivalence and safety can reduce any potential misconceptions and support consistent treatment adherence.

Two of the original draft statements addressed the consideration of cultural background on treatment approaches. While modified asthma management approaches are essential on a wider scale, the voting panel did not find cultural and social aspects relevant in the context of European healthcare systems. This is in contrast to some published evidence; our literature search included several publications reporting cultural and social factors that affect treatment success in different populations around the world^37,41–48^. Additional studies in local populations suggest that feelings of stigma related to asthma can be associated with cultural factors and/or younger age^49,50^. It may be that there is lower awareness of the impact of cultural beliefs and social stigmas in the European countries represented in the survey, compared with other countries represented in the literature. A patient-centric approach based on the 18 consensus statements is likely to be sufficient to guide treatment across a range of patient populations across Europe.

One of the strengths of this programme was the diversity of participants in the voting panel. The SC and voting panel included both HCPs and patient representatives to ensure the patient view was captured. Representatives from five European countries were included. While this distribution is not representative of the full range of primary care practices across Europe, it does reflect viewpoints from a range of different healthcare systems with varying treatment approaches and diverse patient profiles.

Another strength of the modified Delphi approach that was employed in this programme was the structured methodology used to gain consensus, including a high threshold for consensus ( >85%) and a large voting panel. A high level of consensus was achieved on almost all statements voted on, reflecting support more patient involvement. HCPs supported adopting a patient-centric approach to asthma management, despite potential barriers and increased workload that they may face as a result, such as additional training, longer consultations and more resources.

The main limitation of this programme is that while the voting panel was large, the viewpoints of this group may not be generalisable to broader populations. Consensus statements, while based on information in published literature, are not as convincing as empirical evidence, and are subject to the limitations of the modified Delphi approach^24^. Further work must be done to elucidate different approaches to patient-centred asthma care, including best practices and associated outcomes. It is also necessary to develop tools based on the statements and test them across different populations to validate their effectiveness in improving patient-centred management.

Patient-centric initiatives can be implemented in any number of ways and while not all are associated with improved outcomes for patients, a growing number of patient-centred care initiatives have been met with success and serve as case studies for others to follow^51^.

This consensus programme provides a holistic approach, covering four aspects of patient-centred asthma care. It is hoped that this strategy can lead to improved adherence, better asthma control and enhanced patient quality of life, aligning with value-based care principles. Tailoring treatment to individual patient needs and circumstances is essential for achievement of positive long-term outcomes.

In conclusion, this publication provides robust recommendations that may help to guide HCPs to provide holistic, patient-centred asthma care. A collaborative, multidisciplinary approach enables identification and resolution of any issues, supporting individualised care and improving asthma control outcomes. Now that this consensus programme has identified what needs to be done to increase patient involvement in asthma care, a logical next step would be to provide additional guidance on how to put it into practice. Tailoring treatment to individual patient needs and circumstances is essential for achievement of positive long-term outcomes.

## Supplementary information

Supplementary Information

## Data Availability

No datasets were generated or analysed during the current study.
