# Supplementary material for: A guide for a patient-centric approach to asthma management: results of a European Delphi consensus programme
Source: NPJ Prim Care Respir Med. 2025 Dec 19;36:2. doi: 10.1038/s41533-025-00465-3 (PMC12770437; doi:10.1038/s41533-025-00465-3)
Supplement: Supplementary file 1 — Supplementary Information [file 41533_2025_465_MOESM1_ESM.pdf]

## Supplementary materials

**Table S1.** Target HCP profile and screening questionnaire

**Ideal target HCP profile**

- Certified to practice medicine
- Spends at least 70% of time seeing/treating patients
- ≥10 years' experience in managing adult patients with asthma
- ≥10 patients with asthma in monthly review
- Added value if active in publications and research in asthma in the last 5 years, especially if focused on inhaler selection and patient perceptions/preferences

| **Questions/answers** | **Inclusion/exclusion** |
|---|---|
| 1. What is your medical specialty?<br>a. General practice<br>b. Pulmonologist/respiratory physician<br>c. Allergist<br>d. Asthma nurse specialist<br>e. Other: *[free text field]* | **a–d:** Include<br>**e:** Include other relevant specialties if insufficient numbers (to be confirmed by chair) |
| 2. How long have you been practising medicine?<br>a. <3 years<br>b. 3–5 years<br>c. 6–10 years<br>d. 11–20 years<br>e. >20 years | **a and b:** Exclude<br>**c:** Include if numbers are insufficient (to be confirmed by chair)<br>**d and e:** Include |
| 3. What proportion of your time is spent seeing patients per week?<br>a. <20%<br>b. 20–50%<br>c. 51–70%<br>d. >70% | **a and b:** Exclude<br>**c:** Include if numbers are insufficient (to be confirmed by chair)<br>**d:** Include |
| 4. What is the primary setting where you practice?<br>a. General practice community clinic<br>b. Multidisciplinary community clinic<br>c. Specialist respiratory/asthma OR respiratory/asthma community clinic<br>d. General hospital<br>e. Specialist respiratory/asthma OR respiratory/asthma hospital | **a–e:** Include with aim to recruit a mix<br>**f:** Include but deprioritise<br>**g:** Prioritise any mentions of allergy or airway clinics |

| | |
|---|---|
| f. Non-community/private<br>g. Other: *[free text field]* | |
| 5. How many years have you been managing patients with asthma?<br>a. 0–3 years<br>b. 4–7 years<br>c. 8–10 years<br>d. 11–15 years<br>e. 16 or more years | **a and b:** Exclude<br>**c:** Include if insufficient numbers<br>**d and e:** Include |
| 6. How many patients with asthma do you manage on average per month?<br>a. 0–3 patients<br>b. 4–7 patients<br>c. 8–10 patients<br>d. 11–15 patients<br>e. 16 or more patients | **a and b:** Exclude<br>**c:** Include if insufficient numbers<br>**d and e:** Include |
| 7. Have you published, or been involved in, research in asthma in the last 5 years?<br>a. Yes<br>b. No | **a.** Prioritise<br>**b.** Do not prioritise |
| 8. If yes to question 7, was this work focused on inhaler selection and patient perceptions/preferences?<br>a. Yes<br>b. No<br>c. Not applicable | **a.** Prioritise<br>**b.** Do not prioritise<br>**c.** Do not prioritise |

HCP, healthcare professional.

**Table S2.** Target patient profile and screening questionnaire

**Ideal target patient profile**

- Adult patients who live with asthma and use inhaler treatment
- Germany, Italy, France:
  - 12 patients from each country with asthma duration of ≥5 years with the aim to recruit a mix who have:
    - Changed their asthma treatment within the last 5 years (>60%)
    - Received the same asthma treatment for last 5 years (<40%)
  - Aim to recruit, at minimum, 3 out of the 12 patients who have an active role in local patient advocacy group operations and an understanding of the attitudes and perceptions of other patients living with asthma
- Czechia, Greece:
  - 7 patients from each country with asthma duration of ≥5 years with the aim to recruit a mix who have:
    - Changed their asthma treatment within the last 5 years (>60%)
    - Received the same asthma treatment for last 5 years (<40%)
  - Aim to recruit, at minimum, 3 out of 7 patients who have an active role in local patient advocacy group operations and an understanding of the attitudes and perceptions of other patients living with asthma

| **Questions/answers** | **Inclusion/exclusion** |
|---|---|
| 1. What is your age?<br>a. Under 18 years<br>b. 18–24 years<br>c. 25–50 years<br>d. 51+ years | **a:** Exclude<br>**b–d:** Include |
| 2. Have you ever been diagnosed with asthma?<br>a. Yes<br>b. No | **a:** Include<br>**b:** Exclude |
| 3. How many years ago were you diagnosed with asthma?<br>a. 1–2 years ago<br>b. 3–4 years ago<br>c. 5–8 years ago<br>d. More than 8 years ago | **a and b:** Exclude<br>**c and d:** Include |
| 4. If you answered **c** or **d** for question 3, do you currently use an inhaler for your asthma?<br>a. Yes | **a:** Include<br>**b:** Exclude |

| | |
|---|---|
| b. No | |
| 5. If yes to question 4, has your asthma treatment changed within the last 5 years?<br>a. Yes<br>b. No | Include a mix (aim to have >60% patients who answer **a** and >40% patients who answer **b**) |
| 6. Are you part of a patient advocacy group for asthma?<br>a. Yes<br>b. No | Include **a and b** with a minimum of 3 patients who answer **a** (and meet inclusion criteria for questions 3–5) |
| 7. If yes to question 6, do you play an active role in the operations of the group?<br>a. Yes<br>b. No | **a:** Include<br>**b:** Include but deprioritise |
| 8. How would you rate your understanding of the attitudes and perceptions of other patients living with asthma?<br>a. Very good understanding<br>b. Good understanding<br>c. Neutral<br>d. Poor understanding | **a. and b:** Include<br>**c:** Include if numbers are low but deprioritise<br>**d:** Exclude |

**Table S3.** Search criteria used in the non-systematic literature review

| **Search limiters** |
|---|
| • Date: 1 January 2014 to 24 April 2024<br>• Species: Humans<br>• Data type: Applied clinical, any phase, interventional and observational; preclinical and mechanistic excluded<br>• Article language: No filter |
| **PubMed search string** |
| (Asthma[Title/Abstract]) AND (Preference[Title/Abstract] OR “patient preference”[MeSH] OR Perspective[Title/Abstract] OR Belief[Title/Abstract] OR Behaviour[Title/Abstract] OR Behavior[MeSH] OR Attitude[Title/Abstract] OR Attitude[MeSH] OR View[Title/Abstract] OR Fear[Title/Abstract] OR Fear[MeSH] OR Concern[Title/Abstract] OR Anxiety[Title/Abstract] OR Anxiety[meSH] OR Challenge[Title/Abstract] OR Barrier[Title/Abstract] OR Need[Title/Abstract] OR Expectation[Title/Abstract] OR Motivation[MeSH] OR “Shared decision making”[Title/Abstract] OR "Decision Making, Shared"[MeSH] OR Multidisciplinary[Title/Abstract] OR Interdisciplinary[Title/Abstract]) AND (Adherence[Title/Abstract] OR “Medication adherence”[MeSH] OR Persistence[Title/Abstract] OR Inhaler[Title/Abstract] OR Compliance[Title/Abstract] OR “patient compliance”[MeSH]) AND ("2014/01/01"[Date - Publication] : "3000"[Date - Publication]) |
| **Cochrane database search string** |
| (Asthma) AND (Preference OR Perspective OR Belief OR Behaviour OR Attitude OR View OR Fear OR Concern OR Anxiety OR Challenge OR Barrier OR Need OR Expectation OR “Shared decision making” OR Multidisciplinary OR Interdisciplinary OR “Patient care team”) AND (Adherence OR Persistence OR Inhaler OR Compliance) in Title Abstract Keyword - with Cochrane Library publication date Between Jan 2014 and Apr 2024 (Word variations have been searched) |

MeSH, medical subject headings.

**Table S4.** Publications included in final literature review (n=40)

| Reference | Study type | Country | N |
|---|---|---|---|
| **Effect of patient perspectives on inhaler adherence** | | | |
| Axelsson M, et al. The significance of asthma follow-up consultations for adherence to asthma medication, asthma medication beliefs, and asthma control. Nurs Res Pract. 2015;2015:139070.[31] | Cross-sectional survey | West Sweden | 223 |
| Axelsson M, et al. Personality and unachieved treatment goals related to poor adherence to asthma medication in a newly developed adherence questionnaire - a population-based study. Multidiscip Respir Med. 2016;11:42.[29] | Cross-sectional survey | International | 104 |
| Cai Q, et al. Patients’ adherence-related beliefs about inhaled steroids: application of the Chinese version of the Beliefs about Medicines Questionnaire-specific in patients with asthma. J Asthma. 2020;57:319–26.[39] | Cross-sectional survey | China | 217 |
| Chan AHY, et al. SABA Reliance Questionnaire (SRQ): identifying patient beliefs underpinning reliever overreliance in asthma. J Allergy Clin Immunol Pract. 2020;8:3482–9.e1.[45] | Observational | Not reported | 446 |
| Chiu KC, et al. Patients' beliefs and behaviors related to treatment adherence in patients with asthma requiring maintenance treatment in Asia. J Asthma. 2014;51:652–9.[32] | Cross-sectional, observational | China, Korea, Malaysia, Taiwan, Thailand, Vietnam | 1,054 |
| Cooper V, et al. Patient-reported side effects, concerns and adherence to corticosteroid treatment for asthma, and comparison with physician estimates of side-effect prevalence: a UK-wide, cross-sectional study. NPJ Prim Care Respir Med. 2015;25:15026.[46] | Cross-sectional survey | UK | 1,524 |
| Dong R, et al. The association of depressive symptoms and medication adherence in asthma patients: the mediation effect of medication beliefs. Res Social Adm Pharm. 2024;20:335–44.[14] | Cross-sectional survey | China | 399 |
| Fan Q, et al. The mediating role of trust in physician and self-efficacy in understanding medication adherence in severe asthma. Respir Med. 2021;190:106673.[28] | Cross-sectional survey | Singapore | 117 |
| Foot H, et al. Better understanding the influence and complexity of beliefs on medication adherence in asthma. Patient Educ Couns. 2019;102:564–70.[13] | Cross-sectional | Australia | 198 |

| | | | |
|---|---|---|---|
| Heluf H, et al. Factors associated with uncontrolled asthma among adult asthmatic patients in eastern Ethiopia: a multicenter study. SAGE Open Med. 2022;10:20503121221132165.[41] | Observational | Ethiopia | 416 |
| Heluf H, et al. Adherence to anti-asthma medications among adult asthmatic patients in Eastern Ethiopia: a multi-center cross-sectional study. PLoS One. 2022;17:e0277796.[40] | Observational | Ethiopia | 320 |
| Müller S, et al. Identifying the causes increasing the risk of non-adherence in adult patients with asthma: an analysis combining patient survey data with German Claims Data. Drugs Real World Outcomes. 2021;8:207–14.[47] | Multicentre, observational | Germany | 524 |
| Shayo GA, et al. Inhaler non-adherence, associated factors and asthma control among asthma patients in a tertiary level hospital in Tanzania. East Afr Health Res J. 2022;6:78–85.[43] | Observational | Tanzania | 385 |
| Smits D, et al. Factors related to good asthma control using different medical adherence scales in Latvian asthma patients: an observational study. NPJ Prim Care Respir Med. 2017;27:39.[37] | Cross-sectional survey | Latvia | 352 |
| Smits D, et al. Factors related to poor asthma control in Latvian asthma patients between 2013 and 2015. Scand J Prim Health Care. 2017;35:186–91.[36] | Cross-sectional survey | Latvia | 352 |
| Smits D, et al. Factors related to poor adherence in Latvian asthma patients. Allergy Asthma Clin Immunol. 2020;16:16.[48] | Cross-sectional survey | Latvia | 352 |
| Wang KY, et al. Examining the causal model linking health literacy to health outcomes of asthma patients. J Clin Nurs. 2014;23:2031–42.[49] | Cross-sectional survey | Taiwan | 326 |
| Wang J, et al. Determinants of ICS therapy adherence in patients with asthma. Am J Manag Care. 2021;27:e36–41.[50] | Observational | China | 350 |
| **Patient preference for inhaler devices** | | | |
| Alvarez-Gutiérrez FJ, et al. Preference for Easyhaler® over previous dry powder inhalers in asthma patients: results of the DPI PREFER observational study. Patient Prefer Adherence. 2021;15:349–58.[51] | Cross-sectional, observational | Spain | 485 |
| Andres J, et al. [Therapy adherence in bronchial asthma patients: Healthcare under everyday conditions in pulmonological practices]. Pneumologie. 2014;68:315–21.[52] | Qualitative | Germany | 3,293 |

| | | | |
|---|---|---|---|
| Baggott C, et al. Patient preferences for symptom-driven or regular preventer treatment in mild to moderate asthma: findings from the PRACTICAL study, a randomised clinical trial. Eur Respir J. 2020;55:1902073.[6] | Subgroup analysis of RCT | New Zealand | 306 |
| Baggott C, et al. Patient preferences for asthma management: a qualitative study. BMJ Open. 2020;10:e037491.[5] | Qualitative | New Zealand | 27 |
| Balamurugan S, et al. Device-handling study of a novel breath-actuated inhaler, Synchrobreathe®, versus a pMDI. Respir Med. 2020;161:105707.[53] | Prospective, open-label, comparative, multicentre | India | 460 (239 with asthma) |
| Chapman S, et al. Modelling the effect of beliefs about asthma medication and treatment intrusiveness on adherence and preference for once-daily vs. twice-daily medication. NPJ Prim Care Respir Med. 2017;27:61.[54] | Cross-sectional survey | Germany, UK, Spain, France, Italy | 1,010 |
| Contoli M, et al. Exploring quality of life and satisfaction with treatment in asthmatic patients receiving dry powder inhalers: a multinational survey. J Asthma. 2022;59:1473–83.[55] | Survey | Spain | 1,036 |
| Dal Negro RW, Povero M. Acceptability and preference of three inhalation devices assessed by the Handling Questionnaire in asthma and COPD patients. Multidiscip Respir Med. 2016;11:7.[56] | Survey | Italy | 176 |
| Ding B, et al. Maintenance inhaler preference, attribute importance, and satisfaction in prescribing physicians and patients with asthma, COPD, or asthma-COPD overlap syndrome consulting for routine care. Int J Chron Obstruct Pulmon Dis. 2018;13:927–36.[57] | Cross-sectional survey | Europe, USA, Japan, China | 7,305 |
| Giner J, et al. Assessment of inhalation errors, training time and patient preference for DuoResp® Spiromax® and Symbicort® Turbuhaler® in patients with asthma and COPD. Eur Clin Respir J. 2020;8:1833411.[33] | International, multicentre | Spain, Portugal | 161 |
| Israel E, et al. A discrete choice experiment to assess patient preferences for asthma rescue therapy and disease management. J Allergy Clin Immunol Pract. 2023;11:2781–91.[58] | Discrete choice experiment | USA | 1,184 |

| | | | |
|---|---|---|---|
| Jahedi L, et al. Inhaler technique in asthma: how does it relate to patients' preferences and attitudes toward their inhalers? J Aerosol Med Pulm Drug Deliv. 2017;30:42–52.[12] | Qualitative and questionnaire | Australia | 25 |
| Koya T, et al. Influence of adherence to inhaled corticosteroids and inhaler handling errors on asthma control in a Japanese population. 2018;57:3357–63.[38] | Survey | Japan | 290 |
| Nakanishi Y, et al. Association between patient preference for inhaler medications and asthma outcomes. J Asthma Allergy. 2022;15:1539–47.[59] | Observational | Japan | 351 |
| Parimi M, et al. Persistence and adherence to ICS/LABA drugs in UK patients with asthma: a retrospective new-user cohort study. Adv Ther. 2020;37:2916–31.[60] | Retrospective database review | UK | 4,217 |
| Plaza V, et al. Impact of patient satisfaction with his or her inhaler on adherence and asthma control. Allergy Asthma Proc. 2018;39:437–44.[34] | Cross-sectional, multicentre | Spain | 778 |
| Ray SE, et al. Patient perceptions of switching to a generic dry powder inhaler - increased understanding through journey mapping. Int J Chron Obstruct Pulmon Dis. 2022;17:1751–68.[35] | Qualitative | USA | 36 |
| Schreiber J, et al. Inhaler devices in asthma and COPD patients - a prospective cross-sectional study on inhaler preferences and error rates. BMC Pulm Med. 2020;20:222.[61] | Prospective, open-label, cross-sectional | Germany | 105 |
| Tamási L, et al. Clinical effectiveness of budesonide/formoterol fumarate Easyhaler® for patients with poorly controlled obstructive airway disease: a real-world study of patient-reported outcomes. Adv Ther. 2018;35:1140–52.[62] | Real-world, multicentre, open-label, non-randomised, non-interventional | Hungary | 1,498 (621 with asthma) |
| Van der Palen J, et al. A randomised open-label cross-over study of inhaler errors, preference and time to achieve correct inhaler use in patients with COPD or asthma: comparison of ELLIPTA with other inhaler devices. NPJ Prim Care Respir Med. 2017;27:17001.[63] | Multicentre, open-label, randomised, placebo-controlled | UK, Netherlands | 162 |
| Van der Palen J, et al. A randomized, cross-over study comparing critical and overall errors, learning time, and preference of the ELLIPTA versus BREEZHALER dry powder inhalers in patients with asthma. Respir Med. 2022;205:107031.[64] | Multicentre, open-label, placebo-controlled | Netherlands | 114 |

| | | | |
|---|---|---|---|
| Vinge I, et al. A non-interventional switch study in adult patients with asthma or COPD on clinical effectiveness of salmeterol/fluticasone Easyhaler® in routine clinical practice. Ther Adv Respir Dis. 2021;15:175.[65] | Prospective, open-label, multicentre, non-interventional | Germany, Sweden | 231 (178 with asthma) |

COPD, chronic obstructive pulmonary disease; DPI, dry powder inhaler; ICS, inhaled corticosteroids; LABA, long-acting beta agonist; pMDI, pressurised metered-dose inhaler; RCT, randomised controlled trial; SABA, short-acting beta agonist.

**Table S5.** Levels of consensus reached for each statement

| Consensus statements | Level of agreement, % |
|---|---|
| **Patient-centred treatment selection** | |
| Treatment should fit a patient's daily life and be personalised. Problems like cost, trouble using the inhaler and beliefs about asthma or inhalers need to be considered. This will help patients use their inhalers regularly and correctly.[15,47,48,66–68] | 97.50 |
| Doctors/nurses and patients should work together to find out how asthma affects the patient's life. They should also discuss why asthma control is important for their short- and long-term goals of treatment. This will make sure that patients use their inhaler regularly and are happy with it.[52,55,68] | 98.75 |
| Correct inhaler technique and the ability to breathe in strongly are key for deciding between dry powder inhalers (DPIs) and metered-dose inhalers (MDIs). DPIs are not right for patients who can't inhale strongly enough.[3] | 90.00 |
| *[not accepted]* When choosing a treatment, doctors and nurses should remember that some patients may feel embarrassed, face social stigma or have cultural beliefs that affect how they use an inhaler. These factors can affect treatment adherence.[32,47,67–71] | *[not accepted]* 67.82% |
| **Medication and asthma beliefs** | |
| Patients' understanding of asthma and why they need an inhaler can differ. Doctors/nurses should check what patients know about why they need treatment and give them the right information and education if needed. This helps choose the best treatment and encourages regular use.[11,31,32,39,48,68–70,72] | 98.75 |
| What patients know, believe and have experienced about how well asthma inhalers work and how safe they are can affect if they use them as suggested. Doctors/nurses should ask patients to share their thoughts and worries about inhalers, so any confusion or concerns can be cleared up.[43,68] | 93.75 |
| Any reasons why a patient might not follow their treatment should be discussed openly. A team approach helps find and fix any problems, leading to better asthma control.[47,48,68] | 97.50 |
| **Patient preference and shared decision-making** | |
| Including patients in decisions about their asthma treatment can lead to better results.[42,55] It can help them stick to their treatment if it is right for them, and it fits their needs and lifestyle.[11,16,42,52] | 93.75 |

| | |
|---|---|
| How easy the inhaler is to use and the right technique can affect if a patient is satisfied and follows their treatment. Doctors/nurses should provide training and agree with their patient on the right device for them.[12,55,61] | 96.25 |
| Choosing the right inhaler should consider both the medicine and practical things, like portability and a dose counter. This helps make sure the treatment fits the patient's needs in both aspects.[12,55,61] | 95.00 |
| Some patients prefer using just one inhaler instead of two or more. Doctors/nurses should take this into account to plan a patient's treatment. It can help to improve patient satisfaction and make it easier for them to stick to their treatment.[54,55,58] | 90.00 |
| Some patients have concerns about non-branded inhalers compared with brand name ones. Doctors/nurses should address these concerns when they choose a non-branded inhaler to make sure patients follow their treatment.[35] | 85.90 |
| Doctors/nurses should teach their patients about different inhalers, how to use them, and their risks and benefits. This helps to decide on a treatment together and can improve how well patients follow their treatment.[11,72] | 97.50 |
| Doctors/nurses and patients should agree on what good asthma control looks like and how to reach it. This shared understanding of treatment goals helps with better asthma care.[11,16,42] | 97.50 |
| Treatment goals should be reviewed at each visit to make sure both the patient and provider are working towards the same goals. | 98.75 |
| Both the doctor/nurse and patient should agree on a treatment plan. This makes sure the plan meets the patient's needs, leading to better results and regular use of the treatment.[11,16,42] | 92.50 |
| **Utilising tools and technology to support patient-centred care** | |
| Doctors/nurses can use tools like the RACE questionnaire to find out what might make it hard for a patient to stick to their inhaler treatment.[26] | 86.08 |
| There are many apps and tools that can help patients check if they are using their inhaler properly and often enough. Doctors/nurses should assess each individual patient and, if appropriate, suggest apps or tools that might help them. | 91.25 |
| Patients should get treatment or self-management plans in writing, either electronically (so they can print them) or on paper. | 91.25 |

DPI, dry powder inhaler; MDI, metered-dose inhaler; RACE, Respiratory Adherence Care Enhancer.
